# Transforming health care: an approach to system-wide implementation

**DOI:** 10.5334/ijic.1206

**Published:** 2013-09-25

**Authors:** Rafael Bengoa

**Affiliations:** Health Department, Deusto Business School, Bilbao, Spain

**Keywords:** system transformation, population health, integrated care, proactive care, telesilos, triple aim

## Abstract

There are enormous financial, demographic, epidemiological and clinical safety pressures on health care systems around the world. These pressures are well known and are increasing every day.

This perspective paper describes one possible way forward to meeting these pressures undertaken through the system-wide transformation of the health care system in the Basque Country to a population of over 2.3 million people. The overall goal has been to turn the health care system to become more proactive than reactive and more collaborative than fragmented. This ambitious plan started in 2009.

## Context

*Make no little plans. They have no impact to stir men′s blood and probably will not themselves be realized* [1]

To face the significant, growing and long-term pressures on health care systems, it is necessary to have a ‘big’ plan. The Basque public health care system is a single-payer Beveridge type of National Health System. In the Basque health care system, 10% of the population (85% patients with chronic conditions, most of them multi-morbid, complex patients) consume 61% of the financial resources available; 63.55% of the total expenditure is spent on multi-morbid patients and approximately 50% of hospital admissions are non-programmed (non-elective) admissions.

Beveridgian National Health Systems are just as fragmented as any other system when it comes to delivery of care at the grass-roots level. This is not because the single payer National Health System model is flawed. It is because it is not used to its full potential. The reform of the Basque health care system sought precisely that objective, to use the full potential of a single-payer system.

The reform of the Basque health care systems was driven by many projects which were framed as health service research in order to evaluate processes and outcomes and provide evidence for improvements. The main focus of the reform process was to achieve much more collaboration and proactivity in the health care system, and a wide range of tools were initiated and implemented simultaneously.

The reforms of the Basque system followed, to an extent, the logic of the Chronic Care Model [2], but the importance of the approach was how this was explicitly agreed and applied at the policy level to drive forward implementation. In change management terms, we applied four broad lines of work:

- Developing a favourable policy environment
- Stimulating system thinking with new models of care
- Aligning bottom-up and top-down ‘integrators’
- Promoting a distributed leadership approach

## Developing a favourable policy environment

It is widely recognised that pilot experiences in themselves are frequently not expandable or transferable to different contexts and that, therefore, a sufficient scale of change is not reached in a community or a country. Achieving scale, therefore, requires a necessity and a *policy-level intervention* that from the outset provides the health sector with a clear policy that sets the agenda for more proactive and collaborative care. The aim in this case was explicitly raising chronicity to the policy level [3]. The intention was to provide a narrative, one which goes beyond the prevalent cost-containment policies in most of Europe.

Integrated care and chronicity provide a more compelling narrative for why change should be sought. It speaks to an issue which patients and health care professionals can relate to, and therefore, are more ready to adopt. In the Basque system, a key political decision was to consider this transformation process as ‘organic’, implying it needed to be considered as a cultural change. Hence, it was decided not to use a regulatory, legislative approach in the first instance.

## Stimulating system thinking with ‘new’ models of care

The change management agenda were centred on introducing new models of care which helped to create ‘local systems of care’. The models of care that were used were based on the Chronic Care Model [2], the Triple Aim [4]; and risk stratification. These frameworks and models are well known, but the important issue is to signal their relevance in a complex change process since they stimulate ‘system’ thinking and open the door to a population health perspective. Using these frameworks it becomes possible to target interventions and address preferably those more vulnerable patients at risk of future hospitalisations.

Presently the whole population of the Basque Country is stratified according to their risk of hospitalisation (2.2 million people). This does not imply that the risk stratification tool is as yet being used systematically, but it is worthwhile noting that in the microsystems where it is being used, they are becoming a key factor in the redefinition of the work among team members.

## Aligning bottom-up and top-down ‘integrators’

The design of a narrative and the use of the models described above are not enough to change day-to-day clinical practice. It is necessary to provide a battery of management processes to help local managers and health professionals to change practice on the ground.

At the delivery level, the basic reasoning for this was that the Basque health care system did not have the essential elements required for coordinated or integrated care. These factors are well known across care systems internationally [5], and it was clear that few delivery organisations in the Basque Country were using any of the possible care management processes which support integration of care. We therefore needed to develop these care management processes (i.e. ‘integrators’) and ensure that they all had the potential to reinforce integration of care.

**Figure 1.**
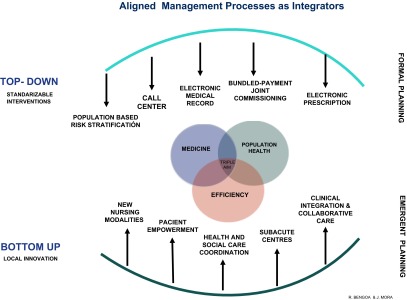
Aligned management processes as integrators

As a first step, the goal was the coordination and integration of organisations at a local level *in clinical terms* rather than a focus on structural or managerial integration. Furthermore the intention was to seek alignment of both top-down and bottom-up ‘integrators’, an alignment based on their integrative potential. Both top-down and bottom-up interventions implemented in the Basque health system since 2009 are described in Figure 1.

*Bottom-up interventions* sought to engage clinical and nursing leadership in the change process. The search for new approaches of clinical leadership requires much greater involvement of health care professionals in the overall effectiveness and performance of the health care system.

*Top-down interventions* followed a more traditional and formal planning approach in view of the fact that they needed to be standardised across the entire health care system. For example, like many other health care systems the Basque resource allocation system was actually financing fragmentation. That is to say, we allocated resources to the providers of the system individually - hospitals, primary care centres, social services - to carry out activity. We were not allocating resources to providers so that they integrated their work. We therefore launched a new approach to joint commissioning (bundled payments across primary and hospital care) geared to encourage coordinated work at the provider level and to incentivise innovation in local care delivery.

## Avoiding ‘Telesilos’

Many of the above changes require the use of digital and information technologies. These are key integrators between the levels of care including home-based telehealth and telecare. However, the technological trend is replicating the existing organisational model of managing one disease at a time and therefore may inadvertently reinforce the silo effect. In a complementary way, technology must also help us manage multiple morbidity in a context in which more than 60% of expenditure is spent on multi-morbid complex patients. This logic was present in the change process of the Basque health system including new payment mechanisms to promote improved delivery of care to meet the needs of people with multiple morbidity.

## Encouraging a distributed leadership approach

One can have a compelling narrative as a policy, interesting new models of care, and a battery of aligned interventions and yet have an inadequate leadership culture. In view of the complexity of the reform process, it was considered unlikely that a traditional management approach which tries to enforce these improvements *onto* the organisation would work.

On the contrary, there is a growing body of evidence indicating that the participation of health care professionals and staff engagement improve both quality and management outcomes in those organisations. Consequently, a major effort was made to encourage a different type of leadership.

It was decided to start with clinical integration based on, among other studies, reviews [6] which suggested that working on clinical integration promotes doctors’ commitment to improvement initiatives. In the first year, 150 bottom-up projects were initiated in this way, a process which further encouraged other health care professionals to come on board.

A secondary advantage of this emergent and local learning approach is that even successful projects can rarely be replicated in other settings. Different groups find different solutions locally as long as they are given the leeway to improve ‘their’ care processes. Based on this premise, scalability of improvement efforts was sought by encouraging self-discovery in all local levels of care rather than transferring successful examples of pilot experiences from one place to another.

A further interesting consequence of launching a bottom-up transformative process is that it reinforced future continuity of projects. In most countries, politicians are changed following elections and subsequently managers as well. If everything has been managed top–down, the probability of those projects fading away with their political promoters is very high. On the contrary, if many projects have been bottom-up they are ‘owned’ locally and will tend to better survive any political turnovers.

## Concluding remarks

The process of transformation of the Basque health care system described in this paper is set in a context of a very deep economic crisis of the country. Simultaneous to this multidimensional reform, it has been necessary to manage the implications of this crisis on health care. That day-to-day crisis management has been centred on taking some major cost-containment decisions especially regarding human resource salaries and the pharmaceutical budget.

The key point to consider in policy terms was to acknowledge that even if these crisis decisions were handled in an effective way they would not transform the health care system to be able to cope with the future challenges of demography, chronicity, fragmentation and sustainability. In other words, it is necessary to understand at the policy level that pure rationing does not deliver the structural changes necessary to the system.

In Europe and elsewhere most of the policy decisions in health care are about having to decide whether to ration or to transform. Rather it is about finding the right balance of both and not letting the first dominate the policy agenda.

## Notes on contributor

Rafael Bengoa is a medical doctor specialised in Management and Community Medicine at the University of London. He has worked extensively both nationally and internationally mainly addressing policy-level reforms in health care. He worked at the World Health Organisation for over 15 years where he was the Director of Health Systems until 2006. From 2009 to 2012 he was the Minister of Health and Consumer Affairs for the Basque Government in Spain. At present he is the Director of the Health Department of the Deusto Business School in Bilbao, Spain, and a Senior Leadership Fellow of the Harvard School of Public Health.
